# Early insights from a national scheme providing vaping devices for smoking cessation: A preliminary evaluation to inform future return‐on‐investment modelling in England

**DOI:** 10.1111/add.70462

**Published:** 2026-05-10

**Authors:** Esther Moore, Duncan Gillespie, Erikas Simonavičius, Leonie Brose

**Affiliations:** ^1^ Sheffield Addictions Research Group, School of Medicine and Population Health The University of Sheffield Sheffield UK; ^2^ National Institute of Health and Care Research (NIHR) Policy Research Unit in Addiction London UK; ^3^ Department of Addictions, Institute of Psychiatry, Psychology and Neuroscience King's College London London UK

**Keywords:** England, e‐cigarettes, health policy, inequalities, quitting, smoking cessation

## Abstract

**Background and aims:**

Swap to Stop is a government scheme to promote smoking cessation. Local authorities in England were given e‐cigarette (vape) starter kits to provide alongside behavioural support in a wide range of settings. This study evaluated (i) scheme uptake by region of England, (ii) proposed delivery settings and the type and length of support, (iii) proposed targeting of priority populations and (iv) product‐cost per 4‐week quit.

**Design, setting and participants:**

From October 2023 to October 2024, local authorities submitted 218 expressions of interest (EOIs) to participate in Swap to Stop. We analysed a sample of 115 (53%) EOIs and associated cost information provided by the Department of Health and Social Care. NHS Quarterly Returns data (April 2024–September 2024) provided 4‐week quit rates.

**Measurements:**

Outcomes were the number of kits requested per adult who smokes in each region of England, the proposed delivery settings and the type and length of support and proposed targeting of priority populations categorised as ‘specific’ (exclusive to certain populations) or ‘targeted’ (accessible to all but targeting particular groups). Product‐cost per 4‐week quit was estimated from the 4‐week quit rates and product cost.

**Findings:**

Regional uptake varied, from 39 kits per 100 people who smoke in the Southwest to 5 per 100 in the Midlands. Most EOIs (75.7%) proposed supplying kits via existing local government funded stop smoking services, followed by physical health care settings (37.4%). A proposed duration of supply of 4 weeks was the most common (48.7%). Thirty‐two percent of the EOIs described specific services, including services exclusively for pregnant women (14.8%) and people experiencing deprivation (13.9%). Frequently targeted were socioeconomically less advantaged groups (e.g. routine/manual workers, 57.4% of EOIs) and people with mental health conditions (36.5% of EOIs). The self‐reported 4‐week quit rate for those who received a kit was 34.3% and the average kit cost was £38.78 (the maximum cost was £40), giving an estimated product‐cost per 4‐week quit of £113.17, not including additional costs incurred by service providers.

**Conclusions:**

Swap to Stop in England, a government scheme to promote smoking cessation through provision of free e‐cigarette starter kits, demonstrated variation in both uptake and the types of services offering free vapes, yet the early quit rates, and consequently the product‐cost per quit, were comparable to those observed in similar smoking cessation schemes. There is potential for further targeting of priority populations with increased investment to ensure people have the best chance of quitting.

## INTRODUCTION

Evidence supports the use of e‐cigarettes (vapes) for smoking cessation. A Cochrane review found high‐certainty evidence that vapes that contain nicotine increase quit rates compared to nicotine replacement therapy (NRT), including in pregnancy [1]. In addition, trial and modelling evidence shows that vapes are cost‐effective compared to standard NRT [2, 3, 4, 5, 6, 7, 8]. A 2018 study ran for just under 3 months and involved giving people who smoked vouchers for a free vape, charger, nicotine liquid and support [2]. The vouchers were advertised via websites and distributed via social housing providers, pharmacies and local government funded community stop smoking services (SSS). Overall, the study reported a 4‐week quit rate of 37%, with the conservative assumption that anyone not followed up was still smoking cigarettes. A feasibility trial of homeless centres, [9] a pilot study of commercial vape shops [6] and a randomised controlled trial of emergency departments, [10] all showed positive results in terms of smoking cessation and acceptability/feasibility.

In 2019, the then United Kingdom (UK) Government set a target for England to be smokefree by 2030, with smokefree being defined as no more than 5% of the population smoking [11]. An independent review published in June 2022 predicted that this target would be missed by 7 years if additional policies were not brought in [12]. This review recommended that all people who smoke cigarettes should be offered vapes to help them quit. In April 2023, the previous UK Government announced the national Swap to Stop Scheme [13]. The scheme allows local authorities (LAs) to submit an expression of interest (EOI) to the Department of Health and Social Care (DHSC). Once approved, LAs received vaping starter kits (i.e. the device and liquid of their choice to provide alongside behavioural support to people 18 years old and over in England who smoke) either directly or via vouchers, which can be redeemed for an equivalent kit, up to a maximum value of £40 [including value‐added tax (VAT)] per kit. An estimated 300 000 free vapes were expected to be delivered via existing local government funded community SSS [14], and an additional 600 000 were expected to be delivered via local partnerships. These partnerships are led by LAs and aim to support services with a high smoking prevalence among users, for example, substance misuse services and social housing groups.

This study is part of a wider evaluation of Swap to Stop [15, 16, 17]. We used data from the first 12 months of Swap to Stop in England (October 2023 – October 2024) to address four objectives: (i) To assess the regional uptake of Swap to Stop and its alignment with areas of high smoking prevalence; (ii) to describe the proposed delivery settings and the type and length of support, (iii) to determine the extent to which the services using Swap to Stop proposed targeting priority smoking populations; and (iv) to estimate the product‐cost per quit. As this study focuses on the EOI process and initial data from Swap to Stop, its purpose is not to provide a final assessment, but to aid future return‐on‐investment modelling and support decisions for future implementation of similar schemes.

## METHODS

### Design

The broader mixed methods evaluation of Swap to Stop is informed by a logic model defining Swap to Stop's intended outputs and outcomes. [15] This article focuses on the return‐on‐investment component of the evaluation, presenting early insights across the process, impact and economic evaluation domains (Table 1). The analysis in this study was conducted without pre‐registration, therefore, the findings should be considered exploratory.

**TABLE 1:** Mapping of the components of this study and data sources to the components of the mixed methods framework.

Component of the mixed methods evaluation protocol [15]	Component of this study	Data source
Process evaluation	The uptake of Swap to Stop by different regions of England	DHSC dataset on the cost and allocation of the vape starter kits, smoking prevalence from the Annual Population Survey via DHSC's fingertips and mid‐year population estimates from the Office for National Statistics.
	The proposed delivery settings and the type and length of support	Expressions of interest submitted by local authorities.
	Proposed targeting of priority populations	Expressions of interest submitted by local authorities.
Impact and economic evaluation	Product‐cost per 4‐week quit (including calculation of 4‐week quit rate)	DHSC dataset on the cost and allocation of the vape starter kits and NHS Quarterly Returns data.

Abbreviations: DHSC, Department of Health and Social Care; NHS, National Health Service.

### Data sources

#### Expressions of interest

Since October 2023, LAs have been able to submit EOIs outlining their plans for how they would use the vape starter kits provided by Swap to Stop. By October 2024, 218 EOIs had been received. We extracted data from all submissions in the first wave (November to December 2023, *n* = 68), and randomly sampled submissions from the subsequent waves (*n* = 22 from January to March 2024, *n* = 25 from April to October 2024) until over 50% of the EOIs had been sampled (*n* = 115 EOIs, 53%). Oversampling early EOIs increases the likelihood of including those with data on quit success already published. EOIs were completed by LAs on a standardised form with free text sections (see a blank copy in the Supporting information). Data extracted into an Excel sheet included the eligible population, targeted smoking populations, service setting(s), type and length of behavioural support and proposed duration of vape supply (i.e. what was advertised by the vape supplier). Table S1 provides a full list of extracted data with descriptions.

#### Cost data associated with each expression of interest (the DHSC dataset)

DHSC provided information on the unit cost of vaping kits requested with each included EOI. This dataset indicates kits allocated, not ordered or used, as service providers were responsible for ordering after DHSC funding allocation.

#### Adult smoking prevalence and population estimates

Adult (18+) current smoking prevalence (2023 1‐year average) for each English region was obtained from the Annual Population Survey via DHSC's Fingertips, [18] a large public health data collection available by different geographical areas. We used 2023 mid‐year population estimates from the Office for National Statistics (ONS) to get the number of adults in each region of England [19].

#### National Health Service (NHS) England Digital Quarterly Returns data

Collected by NHS England Digital from local government commissioned services every 3‐months, the data aggregate for each LA, the number of quit attempts made, individual characteristics (e.g. smoking‐related details), the type of support received and the number that led to 4‐week quits [self‐reported and carbon monoxide (CO) verified] [20]. To support Swap to Stop monitoring and evaluation, new tables were added, summarising for each LA: (i) the number of vape starter kit recipients; (ii) of those who received a kit, the number of those whose quit attempt was recorded by a SSS; and (iii) of those who received a kit, the number of 4‐week quitters (regardless of recorded quit attempt or SSS engagement). All reported quits were self‐declared and not necessarily CO verified. This study used the first 6 months of data from April to September 2024 [21], which included 115 LAs reporting on Swap to Stop. Eight (7.0%) had to be excluded because of reporting a higher number of recorded quit attempts than people who had received starter kits, resulting in a final sample of 107 LAs.

### Evaluation

#### Uptake of swap to stop by different regions of England

To assess variations in uptake, we used the DHSC dataset to map the number of vape starter kits allocated to each region (Northeast, Northwest, Yorkshire and Humber, Midlands, East, Southeast, Southwest and London). We also used data on adult smoking prevalence and the population of each region to calculate the number of starter kits allocated per 100 people who smoke in the population [18]. Although these regional figures provide a high‐level overview, smoking prevalence and kit uptake vary across the constituent LAs within each region. Several services outlined in the EOIs spanned multiple LAs, therefore, it was not possible not possible to accurately assess uptake at the LA level.

#### The proposed delivery settings and the type and length of support

To assess which settings were involved in delivering Swap to Stop, we grouped EOI data on the proposed settings into 10 categories: app only (any app), community SSS, community organisations, homeless shelters, mental healthcare settings, physical healthcare (i.e. non‐mental health specific) settings, substance misuse clinics, social care and workplace (Table S2). To describe how services implemented the delivery of Swap to Stop starter kits, EOI data were categorised into: (i) very brief advice (VBA) or (ii) ongoing behavioural support. VBA encompassed services that explicitly used the term ‘very brief advice’ or its variations, such as ‘brief intervention’ or ‘brief advice.’ VBA is defined as a 30‐second intervention involving asking about smoking, advice on the best cessation methods and facilitation of access to support [22]. Ongoing behavioural support was defined as support comprising of more than one session. We recorded separately the number of weeks for which behavioural support and a vape were provided and calculated the proportion of EOIs with these characteristics. We also report the number of EOIs in our sample, which specified if there was additional funding to continue to supply free vapes/vape liquid to individuals after the initial Swap to Stop supply ended, or whether individuals would have to purchase their own.

#### The proposed targeting of priority populations

We extracted data from the EOIs on our primary populations of interest: maternity, socio‐economic deprivation, mental health and physical health (Table S3). These were based on the subgroups highlighted in the EOIs, informed by the DHSC Swap to Stop team and the CORE20PLUS5 priority groups used to guide NHS planning [23]. Data on the populations outside of these primary subgroups were also extracted. The services through which the vapes were delivered were then categorised into two types: (i) ‘specific’: a service only available to certain populations; and (ii) ‘targeted’: a service accessible to all, but targeting particular populations. For example, a service only available to pregnant women (e.g. within a maternity hospital setting) would be ‘specific’ and a service offered to all people who smoke, but for which the EOI described they would prioritise pregnant women would be ‘targeted’. We counted the number of services that were ‘specific’ or ‘targeted’ for each priority population.

#### Estimated product‐cost per 4‐week quit

Using the DHSC dataset on the vape allocation, we calculated the cost of supply. Using Quarterly Returns [21], we calculated the 4‐week quit rate for all individuals who received a vape starter kit and then separately for the subset whose quit attempt was recorded by a SSS. CIs were calculated for these rates using the Wald method [24]. To estimate the product‐cost per quit, we multiplied the average cost of a Swap to Stop vape starter kit by the number of kits distributed in England, then divided by the number of self‐reported 4‐week quits. An upper‐limit product‐cost per quit was also estimated using the DHSC's maximum allowed kit cost of £40. Because of DHSC‐reported anomalies from initial vape company discounts, the 10th percentile, rather than the lowest cost, was used to determine the lower‐limit product‐cost per quit. This cost excludes local service delivery and behavioural support expenses.

## RESULTS

### Uptake by region

**FIGURE 1:**
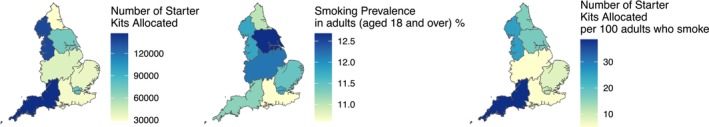
The total number of starter kits allocated, adult smoking prevalence and the number of starter kits allocated per 100 adults who smoke by region in England. [Correction added on 26 May 2026, after first online publication: Figure 1 has been corrected.]

The uptake of Swap to Stop varied across England in both the number of starter kits allocated and the number of starter kits allocated per 100 people who smoke (Figure 1). The Southwest had the largest number of starter kits, with 39 starter kits for every 100 people who smoke, whereas the Midlands had the lowest, with five kits for every 100 people who smoke. Table S4 shows the data used in the calculation.

### Proposed delivery settings and type and length of support

Swap to Stop was mostly proposed to be implemented in community SSS (*n* = 87, 75.7% of EOIs), followed by physical health services (*n* = 43, 37.4%) and maternity services (*n* = 22, 19.1%). Community organisations and other settings such as homeless shelters were less common (Table 2).

**TABLE 2:** The characteristics of the services described in the EOIs.

Characteristic	Number of EOI referenced in (%)
Settings	
Stop smoking services	87 (75.7)
Healthcare settings‐physical	43 (37.4)
Maternity services	22 (19.1)
Substance misuse treatment clinics	16 (13.9)
Workplace	15 (13.0)
Healthcare settings‐mental	14 (12.2)
Community organisations (e.g. charitable groups/community centres)	5 (4.3)
Other (homeless shelters, social care, app only)	10 (8.7)
Type of support	
Ongoing behavioural support	115 (100.0)
Very brief advice^c^	35 (30.4)
Length of ongoing behavioural support	
Less than 12 weeks	11 (9.6)
12 weeks and over^a^	94 (81.7)
Duration of pregnancy + postpartum	5 (4.3)
Proposed duration of Swap to Stop free vape	
2 weeks	6 (5.2)
4 weeks	56 (48.7)
5 weeks	4 (3.5)
8 weeks	8 (7.0)
12 weeks	26 (22.6)
Other (duration of pregnancy, 6‐weeks, 4–8 weeks)	5 (4.3)
Additional vape supply beyond Swap to Stop supply	
Yes	27 (23.5)
No	29 (25.2)
No information	59 (51.3)
Populations prioritised in specific services	
Pregnancy	17 (14.8)
Physical ill health	11 (9.6)
Mental health	11 (9.6)
Substance misuse	10 (8.7)
Experiencing deprivation^b^	8 (7.0)
Social housing tenants^b^	7 (6.1)
Employees of the organisation delivering services.	6 (5.2)
People experiencing homelessness^b^	6 (5.2)
Other (minority groups, social care clients and those involved with the criminal justice system^b^)	3 (2.6)
Populations prioritised in targeted services	
Experiencing deprivation^b^	62 (53.9)
Mental health	42 (36.5)
Physical ill health	36 (31.3)
Pregnancy	32 (27.8)
Social housing tenants^b^	25 (21.7)
Substance use	23 (20.0)
Minority groups (LGBTQ+, ethnic minorities and socially excluded population)	22 (19.1)
People experiencing homelessness^b^	17 (14.8)
Other (those involved in the criminal justice system^b^, social care clients, young adults, employees of organisations delivering services, people vulnerable to fire and the elderly)	10 (8.7)

*Note*: As each EOI could describe more than one service the categories in this table are not necessarily mutually exclusive.

Abbreviations: EOI, expressions of interest; LGBTQ+, lesbian, gay, bisexual, transgender, queer, plus.

^a^ Clients could opt to have less than 12‐weeks of support.

^b^ These populations were defined as experiencing deprivation when looking at the subgroups of interest. See Table S3 for the full definitions.

^c^ Very brief advice also include services that referred to brief interventions, see methods for details.

All EOIs mentioned that at least some of the services through which vapes were offered would offer ongoing behavioural support, mostly lasting at least 12 weeks (*n* = 94, 81.7%). In addition, 35 (30.4%) EOIs described that vapes would also be offered through services that offered VBA only. Most services (*n* = 56, 48.7%) proposed a 4‐week supply of vapes. Generally, proposed durations ranged from 2 to 12 weeks; however, some maternity services proposed offering vapes thoughout the pregnancy. Approximately 50% of EOIs specified whether the individual would have to purchase their own vapes after the Swap to Stop starter kit: 23.5% (*n* = 27) reported additional funding for continued supplies, while 25.2% (*n* = 29) indicated that clients would need to purchase their own (Table 2). Note that percentages do not add up to 100% because categories are not mutually exclusive, as it was possible EOIs described more than one service through which vapes were given out.

### The proposed targeting of priority populations

**FIGURE 2:**
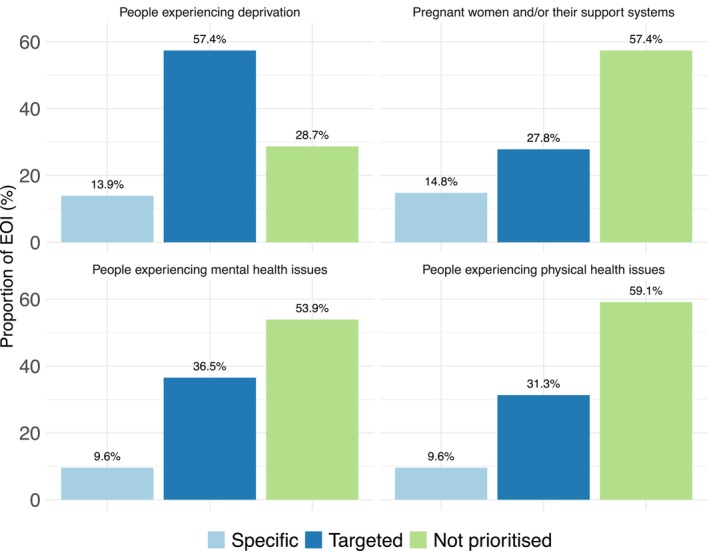
Percentage of expressions of interest (EOIs) prioritising the subgroups of interest through ‘specific’ services (only available to certain populations) or ‘targeted’ services (accessible to all but with stated focus).

Thirty‐seven EOIs (32.2%) had plans to offer a specific service to the primary populations of interest (Figure 2), most commonly for pregnant women and their support systems (*n* = 17, 14.8%), followed by people experiencing deprivation (*n* = 16, 13.9%). Sixty‐nine (60.0%) of EOI proposed targeted services to the primary populations of interest, most commonly people experiencing deprivation (*n* = 66, 57.4%), people with mental health conditions (*n* = 42, 36.5%), people with physical health conditions (*n* = 36, 31.3%) and pregnant women/support systems (*n* = 32, 27.8%) (Figure 2).

Beyond the primary populations of interest, the most prioritised populations for a specific service were those in substance misuse treatment/recovery (*n* = 10, 8.7%) and employees of organisations delivering services (e.g. staff in NHS hospital trusts) (*n* = 6, 5.2%) (Table 2). Of the targeted services, 12 different populations were prioritised in addition to the primary subgroups of interest, most commonly substance misuse (*n* = 23, 20.0%) and minority groups [i.e. lesbian, gay, bisexual, transgender, queer, plus (LGBTQ+), ethnic minorities and socially excluded populations] (*n* = 22, 19.1.0%) (Table 2). As above, categories are not mutually exclusive and one EOI could describe multiple services.

### Estimated product‐cost per 4‐week quit

**FIGURE 3:**
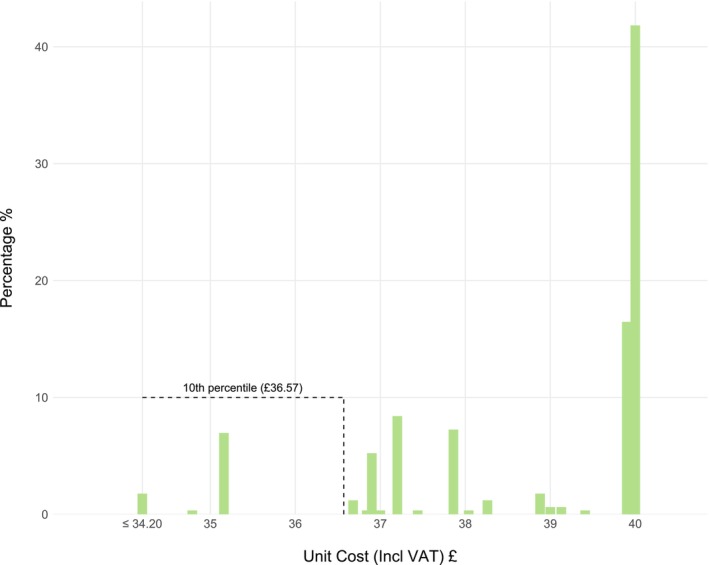
Percentage of expression of interest (EOI) as a function of unit cost.

The unit cost of supply ranged from £23.60 to £40, with a mean of £38.78 (standard error = 0.11) and a mode of £40.00. Figure 3 shows units cost including VAT of each allocation and the percentage of allocations at that unit cost.

From April to September, Quarterly Returns recorded 29 844 people as having received a vape as part of Swap to Stop and 10 227 4‐week quits, giving a 4‐week quit rate of 34.3% (95% CI = 33.7%–34.8%) and a 53.7% (95% CI = 53.0%–54.4%) 4‐week quit rate for those whose quit attempt was recorded by the SSS (Table 3). Using the average cost of supply (£38.78), the overall product‐cost per 4‐week quit was £113.17. Assuming the maximum £40 for all recipients of the starter kit, it increased to £116.73, and assuming the 10th percentile (£36.57), it reduced to £106.71.

**TABLE 3:** Summary of NHS England Digital Quarterly Returns data on local stop smoking Services in England, the number of vapes provided through swap to stop and quit rates.

	*n*	Denominator = number of recipients		Denominator = number of who initiated a recorded quit attempt	
		Quit rate (%)	95% CI	Quit rate (%)	95% CI
Recipients of starter kit	29 844				
Initiated a recorded quit attempt^a^	17 960	60.2%	59.6%–60.7%		
Quit	9647	32.3%	31.8%–32.9%	53.7%	53.0%–54.4%
Unengaged quit^b^	580	1.9%	1.8%–2.1%		
Total quits (engaged + unengaged)	10 227	34.3%	33.7%–34.8%		

Abbreviations: NHS, The National Health Service; 95% CI, 95% confidence interval.

^a^ May include persons that did not engage with multi‐sessional support as part of Swap to Stop.

^b^ Persons that did not engage with multi‐sessional support and/or did not make a recorded quit attempt but who had successfully quit as part of Swap to Stop.

## DISCUSSION

Swap to Stop is the first of its kind, providing vape starter kits or vouchers to public services across England for distribution to people who smoke. Variations in early uptake were observed across the regions of England, as well as in the types of proposed delivery settings and prioritised population groups. Most EOIs in our sample came from community SSS and healthcare providers that tended to have a general population remit while prioritising certain population groups, while more specialised services and services new to providing smoking cessation were less represented. Although all services intended to supply access to behavioural support alongside providing the vape starter kit, in some places it was also possible that recipients would receive only VBA. Among those who received a Swap to Stop starter kit from April to September 2024, 343 4‐week quits per 1000 kits distributed were reported.

The early quit rate (34.3%) is slightly lower than those seen in similar studies (37%– 42%) [2, 6] and in the vape arm of a randomised trial within community SSS in England (43.8%) [25]. However, it exceeds the 30% observed in the NRT arm of this trail [25]. The lower quit rate could be because of the inclusion of people who were given a vape kit toward the end of the quarter, whose outcome will not be captured until the next quarterly data release and the more diverse settings and less controlled intervention. Although none of the studies of similar schemes included a standalone product‐cost per quit calculation, [2, 5, 6, 7, 9] one study did include a total delivery cost‐per‐quit (including both product and staff costs) of £244.70 at 1‐month [5]. There is data available from the NHS Quarterly Returns on the quit rates of other smoking cessation aids, however, this is restricted to individuals who recorded a quit attempt [26]. Because the aim of Swap to Stop is to reach people who are less likely to quit, and recipients did not necessarily have to record a quit attempt, it is not possible to make direct comparisons based on the aggregate summary data in the Quarterly Returns. Individual‐level data from the services is required to directly compare quit rates across different subgroups. If this were available, then the statistical estimates of the effect of providing vape starter kits for different population groups could be used to inform detailed cost‐effectiveness modelling and comparisons of Swap to Stop to alternative programmes.

In addition to the costs of the vaping products and behavioural support, a key determinant of the cost per 4‐week quit and overall cost‐effectiveness is how many people participate (swap) and succeed in quitting (stop). Populations targeted by Swap to Stop may have lower quit rates than those who would have quit using other methods. Previous research has shown that those experiencing socio‐economic disadvantage have lower quits rates compared to their more affluent counterparts [27, 28] likely because of multiple factors including lack of social support, higher levels of dependence, lower motivation to stop smoking and higher exposure to stress [29]. A review found the evidence on whether vapes increase the quit rates in those experiencing socio‐economic disadvantage to be unclear [30]. However, this review did find evidence that vapes can be used to achieve higher or equivalent quit rates to standard care (such as NRT or referral to a SSS) in other priority populations and settings, including pregnant women, [31] people with mental health conditions [32] and services provided within emergency departments [10] or GP surgeries [33]. The evidence for other priority groups and settings such as the LGBTQ+ community and people in homeless shelters and prisons is less clear [30]. To help increase the chance that receiving a vape leads to a successful quit, service commissioners should ensure there is adequate ongoing support to instigate and maintain a smoking quit attempt alongside the vape supply, which includes thinking about how to engage people in this support.

Evidence suggests that standard SSS can act to increase socio‐‐economic inequalities in smoking rates if they take a non‐targeted approach because the people most likely to engage with these services tend to be relatively less vulnerable and have higher quit rates [27]. Vapes could play an important role in reaching other populations, particularly by motivating people who had not yet considered making a quit attempt (i.e. increasing the number of people swapping). A study, part of the wider evaluation of Swap to Stop, used data from a large monthly cross‐sectional population survey and reported that the introduction of the scheme was associated with a 1.5 absolute percentage point increase in the proportion of people in England using vapes in the past 12‐months to aid a quit attempt [15, 16]. Even if people do not stop initially, dual users have been found to be more likely to go on to subsequently quit cigarettes compared to those who only use tobacco cigarettes [34]. Alternative population‐wide schemes, implemented in other jurisdictions, include the distribution of free NRT by post [35, 36]. However, although these schemes were advertised widely, they relied on the individual requesting the offer (i.e. opting‐in). Swap to Stop differs because it aims to deliver the vapes via services that underserved populations might interact with already and uses a more popular cessation aid, with the offer of a free vape providing an opportunity to have an initial conversation about stopping smoking.

The distribution of free vaping starter kits could also reduce the financial burden to the individual associated with the start of a quit attempt. Swap to Stop was introduced at a time when access to vapes was becoming more restricted because of concerns about the health and environmental effects of increased vaping among people who have never smoked, particularly young people. A ban on disposable vapes was implemented in June 2025 [37] and the Tobacco and Vapes Bill includes powers to restrict the availability of vapes, regulate flavours, packaging and display of vapes and bans the advertising of vapes [38]. Additionally, a new vaping duty introduced in October 2026 may make vapes less affordable [39]. Evidence suggests price increases could significantly reduce uptake of vapes, [40, 41, 42, 43, 44] potentially causing people to not use them to support quitting smoking. The free vape provision through Swap to Stop may increasingly function as a financial incentive for people who smoke to quit and promote the use of regulated vaping products from trusted sources. To maximise the health benefits of the scheme and others like it, future work should focus on three areas: (i) how the provision of free vapes can effectively reach individuals who smoke in priority populations who might not usually engage with public services; (ii) how the offer of a free vape can best be used to instigate conversations about stopping smoking; and (iii) how this provision can be accompanied by behavioural support for stopping smoking that is tailored to suit their individual needs.

The success of a scheme such as Swap to Stop in other jurisdictions likely depends on a combination of supportive regulation, established infrastructure for delivering smoking cessation services and the cultural acceptance of vapes for smoking cessation. The UK government takes an evidence‐based approach to the regulation of vapes, [30, 45] aiming to balance the benefits of vapes for smoking cessation with the risk they pose to young people. For example, the Tobacco and Related Products Regulations 2016 introduced restrictions on the marketing of vapes toward children, which will be further strengthened by the Tobacco and Vapes Bill [38] and the National Institute for Health and Care Excellence (NICE) recommends that vapes should be offered as a first‐line cessation aid [46]. This approach to regulation has likely contributed to the population of the United Kingdom having a more evidence‐consistent perception of harm toward vapes compared to countries such as Australia, where vapes are only available via prescription [47, 48]. Other jurisdictions should consider these factors when looking to implement similar schemes.

This study's strengths lie in its direct use of data extracted from the EOIs submitted by participating services and from the official quarterly outcomes monitoring data, which allowed rapid insight into the delivery of Swap to Stop and its outcomes. However, there are four key limitations. First, the analysis was based only on a sample of EOI, oversampling early adopters; later applications may reflect changed requests by these early adopters and different types of requests from later‐adopters that had more time (e.g. to set up partnerships with services that do not typically offer smoking cessation support). Second, we could not assess characteristics of people who received a vape starter kit or whether people were using the vapes alongside cigarettes or other cessation aids, preventing an assessment of effects across different demographics. Further analysis, planned as part of the wider evaluation of Swap to Stop, may reveal differences across subgroups [15]. Third, the EOI outline service plans, but do not tell us about the actual implementation. Fourth, product‐cost per quit provides a limited insight into the return‐on‐investment, as it does not include all costs (e.g. delivery costs) or the long‐term benefits associated with a quit, however, it will be useful in future return‐on‐investment modelling. Determining the cost‐effectiveness is limited by the absence of a counterfactual. Swap to Stop may attract people who would not have otherwise made a quit attempt, however, it may also attract people who would have gone on to quit using other methods.

## CONCLUSION

Swap to Stop in England demonstrated significant variation in the types of services offering free vapes, yet the early quit rates and consequently the product‐cost per quit, were comparable to those observed in similar schemes. There is potential for further targeting of free vapes to priority groups, however, this should be accompanied by targeted support designed to overcome the lower quit rates often seen in these populations.

## AUTHOR CONTRIBUTIONS

**Esther Moore:** Conceptualization; methodology; writing—original draft; writing—review and editing; investigation; formal analysis; data curation; visualization. **Duncan Gillespie:** Conceptualization; methodology; writing—original draft; writing—review and editing; supervision. **Erikas Simonavičius:** Conceptualization; methodology; writing—review and editing. **Leonie Brose:** Conceptualization; methodology; writing—review and editing; supervision.

## DECLARATION OF INTERESTS

None.

## Supporting information


**Table S1** The information extracted from each expression of interest form
**Table S2.** The settings involved in the Swap to Stop scheme
**Table S3.** The definitions of our priority population groups
**Table S4.** Data provided by the Office of Health and Disparities regarding orders for starter kits and NHS England Digital quarterly data on Local Stop Smoking Services in England, detailing vape starter kit orders submitted by local authorities through the Swap to Stop scheme, for each region of England.

## Data Availability

The data from the Expressions of Interests and the data on the number and costs of vaping starter kits requested from the Department of Health and Social Care (DHSC) by individual services were provided for this study in confidence by the DHSC. Data on the adult smoking prevalence is openly available from the Annual Population Survey via https://fingertips.phe.org.uk/ and the data on the number of people who received a vape via the Swap to Stop scheme and those who reported quitting is openly available via NHS England Digital at https://digital.nhs.uk/data-and-information/publications/statistical/statistics-on-nhs-stop-smoking-services-in-england/april-2024-to-december-2024-q3.
