# Supplementary material for: Early insights from a national scheme providing vaping devices for smoking cessation: A preliminary evaluation to inform future return‐on‐investment modelling in England
Source: Addiction. 2026 May 10;121(9):2373–83. doi: 10.1111/add.70462 (PMC13486939; doi:10.1111/add.70462)
Supplement: Supplementary file 1 [file ADD-121-2373-s001.docx]

Supplement

**Article:** Early insights from a national scheme providing vaping devices for smoking cessation: A preliminary evaluation to inform future return‐on‐investment modelling in England

Contents

[Blank expression of interest (EOI) form	1](#_Toc228191671)

[Table S1 The information extracted from each expression of interest form	3](#_Toc228191672)

[Table S2 The settings involved in the Swap to Stop scheme	5](#_Toc228191673)

[Table S3 The definitions of our priority population groups	5](#_Toc228191674)

[Table S4 Data provided by the Office of Health and Disparities regarding orders for starter kits and NHS England quarterly data on Local Stop Smoking Services in England, detailing vape starter kit orders submitted by local authorities through the Swap to Stop scheme, for each region of England.	7](#_Toc228191675)

### Blank expression of interest (EOI) form

**Section 1: The project**

EOI are invited from local authorities and local authority-led partnerships.

| **1.1 Local authority contact details** | | |
| --- | --- | --- |
| **Name of local authority submitting the EOI** (or lead-LA on behalf of partnerships applications) |  | |
| **Are you submitting this EOI on behalf of your SSS alone, or the SSS plus wider partnership organisations?** | ☐ Stop Smoking Service alone | ☐ SSS plus wider LA-led Partnership |
| **Name of Designated Project Lead** |  | |
| **Job title / position in authority** |  | |
| **Telephone number** |  | |
| **Work address and postcode** |  | |
| **Email address** |  | |

If applicable:

**1.2 LA-led partnership details**

**Please list the names of all organisations involved in delivering the project**

**1.3 Project details**

**1.3.1 Please provide details of the project you are proposing (500 words maximum)**

*Include reference to the intervention model offered, target populations and settings, level of need, and readiness to deliver. Note any existing/previous vape offers and how this project will contribute further.*

**1.3.2 Eligibility criteria – provision of behavioural support. (250 words maximum)**

*Please describe the nature of the behavioural support that will be provided and how it will be quality assured.*

**1.3.3 Eligibility criteria – recording activity within local stop smoking service data returns. (250 words maximum)**

*Please confirm arrangements for the accurate and timely recording of activity within the LSSS data returns.*

**1.3.4 Please confirm the target population(s) and how you plan to engage with them. (250 words maximum)**

*Include estimates of local smoking prevalence/volumes within target population(s)*

**1.3.5 Please describe the local governance arrangements for the project. (500 words maximum)**

*Include the arrangements for local oversight and local monitoring of project delivery and how risks will be identified and mitigated (including fraud risk assessment).*

**1.3.6 Please confirm a commitment to work with the national programme evaluators and to disseminate local findings more widely. (250 words maximum)**

**Section 2: Choice and number of vape starter kits**

Include details of the vape starter kits required, as identified on the CCS Tail Spend Vaping Solution platforms. See Annex II of the EOI guidance notes.

| **2.1 Summary of vapes starter kit provider** | | | | |
| --- | --- | --- | --- | --- |
| **Chosen Tail Spend Vaping Solution provider** (you may select more than one) | ☐ OTG | | ☐ Unite (Mercateo) | |
| **Chosen Vendor (vape supplier)** |  | | | |
| **Number of vape starter kit required in this application**  Note: additional applications can be made. | **In 2023/24** | **In 2024/25** | | **TOTAL** |
|  |  |  | |  |

**2.2 Details of vape starter kits required**

**Provide product details of the vape starter kits selected, including unit cost and total costs.** *Note:* *disposable products will* ***not*** *be permitted.*

**2.3 Rationale for choice**

**Please provide a brief rational for your choice of products / vendor (max 250 words)**

*For example, include any local policy positions that shaped your decisions (e.g.* [*Article 5.3*](https://www.gov.uk/government/publications/protocol-for-engagement-with-stakeholders-with-links-to-the-tobacco-industry/guidance-for-government-engagement-with-the-tobacco-industry)*,* [*subsidy assessment*](https://www.gov.uk/government/publications/uk-subsidy-control-statutory-guidance)*).*

**Section 3: Additional Information**

**3. Additional information**

**Section 4: Signatures**

| **4. Local authority Director of Public Health** | |
| --- | --- |
| **Signed** |  |
| **Name** |  |
| **Email** |  |
| **Telephone number** |  |

### Table S1 The information extracted from each expression of interest form

| **Data field(s)** | **Description** |
| --- | --- |
| SSS or local partnership | This indicates whether the proposed service solely involved a stop smoking service or whether it was a local partnership |
| Local authority | These are the local authorities involved in the application |
| Existing service | Indicates whether an existing stop smoking service is in place |
| Information about the existing service | Information includes whether the following were offered:   - Behavioural support - NRT - Other treatment - E-cigarettes |
| Populations eligible for the proposed service | Indicates the populations which are eligible for the service or whether it is a universal offering. |
| Populations prioritised for the service | Indicates priority groups within the eligible population. |
| Referral routes | Indicates how people will be able to access the service. |
| Settings | The organisations/environments where the service is set. |
| Delivery | The staff who will deliver the service |
| Type of behavioural support | Details on the behavioural support including whether its ongoing support, a very brief intervention or self-quit. |
| Length of behavioural support | If the service provides behavioural support this will indicate how many weeks this is for. |
| Vape offer | This indicates whether the proposed offer of vapes is completely new, an expansion generally (adding capacity to existing service) or expansion to specific populations. Also includes the proposed duration of supply. |
| Training | Indicating whether the staff delivering the programme have/will receive specific training on the use of e-cigarettes for smoking cessation. |
| Carbon monoxide | Indicates whether carbon monoxide monitoring or validation will take place. |
| Maintenance | Information regarding what will happen after the Stop to Swap starter kit has finished. |
| Follow-up | Indicates when clients will be followed-up |
| Supply | Supply of vapes directly or via voucher, and if voucher, how these will be delivered to clients (i.e., home delivery, staff order or local shop) |

### Table S2 The settings involved in the Swap to Stop scheme

| Category | The settings mentioned in the expressions of interest |
| --- | --- |
| Community Stop Smoking Services | Community stop smoking services commissioned by the local authority. |
| Healthcare settings- physical | Hospital- outpatients  Hospital- inpatients  Community pharmacy  Emergency departments  Respiratory clinics  GP surgeries  Social prescribing service |
| Healthcare settings- mental | Mental health hospital  Community mental health services |
| Maternity services | Maternity services within a hospital  Maternity services in the community. |
| Substance misuse clinics | Substance misuse clinics |
| Homeless shelters | Rough sleeper centres/homeless shelters |
| Workplace | Job Centre  Workplace  Military |
| Social care | Social care services |
| Community | Community settings e.g., charitable groups |
| Online only | Delivered via apps or social media groups |

### Table S3 The definitions of our priority population groups

| Category | The population mentioned in the expressions of interest |
| --- | --- |
| Maternity | Pregnant women and their support systems |
| Deprivation | Routine/Manual workers  Living in area of deprivation  People experiencing homelessness  Living in social housing  Involved in the criminal justice system  Experiencing multiple disadvantage  Unemployed  Renters  Low income |
| Mental Health | Experiencing any type of mental illness |
| Physical health | Hospital Inpatients  Eligible for lung cancer/health screening  Chronic obstructive pulmonary/respiratory disease  Long-term health condition  Risk of or have cardiovascular disease  Disabled  Awaiting elective surgery  Multiple unhealthy behaviours  Outpatients  Smoking related disease  Cancer patients |

### Table S4 Data provided by the Office of Health and Disparities regarding orders for starter kits and NHS England quarterly data on Local Stop Smoking Services in England, detailing vape starter kit orders submitted by local authorities through the Swap to Stop scheme, for each region of England.

| Region | Number of people who received a vape starter kit | Number of people who set a quit date | Quit attempt rate %* | Number of people who were engaged in a service quit | Number of people who quit but were unengaged with a service | Number of people who quit | 4-week quit rate %^±^ | Total number of starts kits ordered | Number of adults (18+) who currently smoke (2023) | Number of kits ordered per 100 people who smoke |
| --- | --- | --- | --- | --- | --- | --- | --- | --- | --- | --- |
|  |  |  |  |  |  |  |  |  |  |  |
| East of England | 1324 | 1309 | 98.9 | 676 | 0 | 676 | 51.1 | 36580 | 4369501 | 8.4 |
| London | 3069 | 2777 | 90.5 | 1397 | 67 | 1464 | 47.7 | 77618 | 699239 | 11.1 |
| Midlands | 2333 | 2066 | 88.6 | 1210 | 155 | 1365 | 58.5 | 41410 | 804406 | 5.1 |
| North East | 1151 | 984 | 85.5 | 490 | 99 | 589 | 51.2 | 29020 | 177644 | 16.3 |
| North West | 2486 | 1844 | 74.2 | 841 | 146 | 987 | 39.7 | 136769 | 539165 | 25.4 |
| South East | 2209 | 1967 | 89 | 950 | 4 | 954 | 43.2 | 32731 | 596434 | 5.5 |
| South West | 15558 | 5774 | 37.1 | 3553 | 83 | 3636 | 23.4 | 146920 | 381391 | 38.5 |
| Yorkshire and Humber | 1714 | 1239 | 72.3 | 530 | 26 | 556 | 32.4 | 73250 | 424993 | 17.2 |
| **England** | 29844 | 17960 | 60.2 | 9647 | 580 | 10227 | 34.3 | 574298 | 4060223 | 14.1 |

^*^ The percentage of those who received a vape kit who set a quit date

^±^The percentage of those who had received a vape quit and self-reported a 4-week quit
